# Integrating exercise and nutrition to combat frailty: a systematic review of multidomain interventions in aging populations

**DOI:** 10.3389/fnut.2026.1883888

**Published:** 2026-08-05

**Authors:** Guangquan Ren, Zhicheng Zhang, Bo Nan

**Affiliations:** 1 International Football Education School of Jilin Agricultural University, Changchun, Jilin, China; 2 College of Food Science and Engineering, Jilin Agricultural University, Changchun, Jilin, China

**Keywords:** exercise intervention, frailty, geriatric rehabilitation, multidomain intervention, nutritional supplementation, older adults, physical function, resistance training

## Abstract

**Background:**

Frailty is a common geriatric syndrome associated with increased disability, hospitalization, and mortality. Exercise, nutritional supplementation (including herbal supplements such as ginseng), and multidomain interventions have been investigated as strategies to improve frailty-related outcomes in older adults.

**Objective:**

To summarize randomized controlled trial evidence on the effects of exercise, nutritional, and multidomain interventions on frailty, muscle strength, and physical performance in older adults.

**Methods:**

A narrative systematic review of randomized controlled trials (RCTs) involving frail or pre-frail older adults was conducted. Interventions included exercise training, nutritional supplementation (e.g., protein, amino acids, vitamin D, and ginseng-containing nutritional strategies), and combined multidomain strategies. Outcomes included frailty status, gait speed, muscle strength, body composition, cognition, and quality of life.

**Results:**

Exercise-based interventions consistently improved muscle strength, gait speed, and frailty status. Nutritional supplementation alone showed modest and inconsistent benefits, mainly in undernourished or sarcopenic populations. Combined exercise and nutrition interventions generally produced greater improvements in muscle mass and strength than single interventions alone. Multidomain interventions incorporating exercise, nutrition, and cognitive training demonstrated additional benefits in physical and cognitive outcomes, although results varied across studies. Evidence for pharmacologic or anabolic adjuncts remained limited and inconsistent.

**Conclusion:**

Exercise-based interventions, particularly multicomponent resistance and functional training, provide the most consistent benefits for frailty-related outcomes in older adults. Nutritional support may enhance exercise-induced improvements but is insufficient alone to reverse frailty. Multidomain interventions appear promising, though further large-scale standardized trials are needed to determine optimal intervention strategies and long-term effects.

## Introduction

Frailty is a multidimensional geriatric syndrome characterized by reduced physiological reserve and increased vulnerability to stressors, leading to higher risks of falls, hospitalization, disability, and mortality (1, 2). It is commonly operationalized using phenotype-based criteria (e.g., weight loss, exhaustion, weakness, slow gait, low physical activity) or deficit accumulation approaches such as the Frailty Index (1, 2). Among community-dwelling and clinical older populations, frailty overlaps strongly with sarcopenia, malnutrition, and chronic inflammation, forming a clinically important cluster that drives functional decline and loss of independence (3–5). Furthermore, shared mechanisms including anabolic resistance, mitochondrial dysfunction, endocrine dysregulation, and chronic inflammation are thought to underpin the progression of both frailty and sarcopenia (3–5).

Given its reversible or at least modifiable nature in early stages, frailty has become a key target for intervention (6). Exercise-based strategies, particularly resistance and multicomponent training, are widely recognized as the cornerstone for improving muscle strength and physical performance (7, 8). Randomized controlled trials (RCTs) in frail and pre-frail older adults consistently demonstrate that progressive resistance training and multicomponent exercise can improve gait speed, muscle strength, and overall frailty status, and in some cases reverse frailty phenotype classification (9, 10). However, the magnitude of benefit varies depending on adherence, baseline functional status, and intervention intensity.

Nutritional interventions have also been extensively investigated, particularly protein supplementation and amino acid-based strategies aimed at counteracting anabolic resistance in aging muscle (11). Evidence from randomized trials indicates that higher protein intake (e.g., up to 1.5 g/kg/day) can improve appendicular lean mass and gait speed in frail or malnourished older adults (12), while whey protein and oral nutrition supplements can improve strength recovery and rehabilitation outcomes in clinical populations (13, 14). Mechanistically, these interventions are thought to enhance muscle protein synthesis and support recovery from catabolic stress, particularly when combined with exercise training.

Beyond protein, several bioactive nutritional compounds have been studied in frailty-related outcomes, including branched-chain amino acids (BCAAs), beta-hydroxy-beta-methylbutyrate (HMB), omega-3 fatty acids, and medium-chain triglycerides (MCTs) (15, 16). Trials suggest that these interventions may improve muscle function, metabolic signaling, or inflammatory status, although effects on clinically meaningful endpoints such as mobility and frailty reversal are inconsistent (17–20). Importantly, several studies highlight that improvements in strength or biomarkers do not always translate into functional gains, underscoring the complexity of frailty as a systemic condition.

Multidomain interventions combining exercise, nutritional supplementation, and sometimes cognitive or psychosocial components have been increasingly studied as a holistic approach to frailty. Randomized trials and secondary analyses indicate that such interventions can improve gait speed, cognitive function, depressive symptoms, and frailty indices more effectively than single-domain interventions in some populations (21–23). These findings support the concept that frailty is driven by interacting physiological and behavioral domains, and therefore may require integrated interventions to achieve sustained improvement.

Despite a growing body of RCTs, the evidence base remains heterogeneous in terms of populations (community-dwelling vs. institutionalized vs. disease-specific), intervention components, and outcome measures (e.g., gait speed, frailty index, muscle mass, cognitive scores). Moreover, some trials report null or mixed effects, particularly for supplementation alone or in highly frail clinical populations (14, 24). This variability raises uncertainty regarding the overall effectiveness and generalizability of different intervention strategies.

Therefore, this systematic review synthesizes randomized controlled trial evidence on exercise, nutritional, and multidomain interventions targeting frailty and pre-frailty in older adults. The review focuses on clinically relevant outcomes including frailty status, muscle strength, physical performance, and functional independence, with additional consideration of cognitive and metabolic outcomes where available. The aim is to clarify which intervention strategies demonstrate the most consistent benefit and to assess the overall certainty of evidence supporting their use in frailty prevention and reversal.

## Methods

### Study design

This study was conducted as a systematic review of RCTs and controlled clinical studies evaluating the effects of exercise, nutritional supplementation, and combined multidomain interventions on frailty, sarcopenia-related outcomes, and physical or cognitive function in older adults. The review followed a structured evidence synthesis approach consistent with Preferred Reporting Items for Systematic Reviews and Meta-Analyses (PRISMA) methodology principles, including systematic identification, screening, eligibility assessment, and qualitative synthesis of evidence (25).

### Eligibility criteria

Studies were eligible for inclusion if they met the following criteria:

#### Population

Community-dwelling, institutionalized, or clinical populations of older adults (typically ≥65 years) who were classified as frail, prefrail, sarcopenic, or at increased risk of frailty or sarcopenia according to predefined clinical or research criteria were eligible for inclusion. Frailty was identified using validated instruments such as the Fried frailty phenotype, Frailty Index, Edmonton Frail Scale, gait speed thresholds, Short Physical Performance Battery (SPPB), Timed Up and Go (TUG), or disease-specific indices (e.g., Liver Frailty Index (LFI) in cirrhosis populations). Participants considered at risk of frailty or sarcopenia included those with reduced physical performance, low muscle strength, low muscle mass, malnutrition, recent functional decline, mobility limitations, chronic disease–related vulnerability, or other recognized risk factors used by study investigators. Studies enrolling robust older adults without frailty-, prefrailty-, or sarcopenia-related risk characteristics were excluded.

#### Interventions

Eligible interventions included:

- Exercise-based interventions (e.g., resistance training, multicomponent exercise, functional training, aerobic exercise, balance training, or structured physical activity programs).
- Nutritional supplementation interventions (e.g., protein supplementation, amino acids and BCAAs, whey protein, leucine, creatine, HMB, vitamin D, omega-3 fatty acids, MCTs, or oral energy/nutritional supplements).
- Combined exercise–nutrition interventions integrating exercise with one or more nutritional strategies.
- Multidomain interventions combining exercise and nutrition with additional cognitive, educational, behavioral, psychosocial, or lifestyle components.

#### Comparators

Usual care, placebo, standard exercise, standard nutrition advice, or alternative intervention arms.

#### Outcomes

Primary outcomes included frailty status or frailty score changes. Secondary outcomes included muscle strength (e.g., grip strength, knee extension strength), physical performance (gait speed, SPPB, TUG), body composition (lean mass or skeletal muscle index), cognitive outcomes, biomarkers (inflammatory, anabolic, oxidative stress markers), and adverse events.

#### Study design

RCTs, cluster RCTs, and controlled clinical trials were included. Protocol-only studies were excluded from outcome synthesis but noted descriptively where relevant.

### Information sources and search strategy

We searched PubMed/MEDLINE, Scopus, Web of Science, and the Cochrane Central Register of Controlled Trials (CENTRAL) from database inception through December 2025. The final search was conducted on 30 January 2026. Search strategies combined controlled vocabulary terms (e.g., MeSH in PubMed) with free-text keywords across four concept domains: (1) frailty and prefrailty, (2) older adults, (3) exercise and physical training, and (4) nutrition, protein supplementation, and multidomain interventions. Boolean operators were used to combine terms within each domain (“OR”) and across domains (“AND”). Search strategies were adapted to the indexing systems and syntax requirements of each database.

An example PubMed/MEDLINE strategy was as follows:

(“Frailty”[Mesh] OR frailty [tiab] OR frail*[tiab] OR prefrail*[tiab] OR pre-frail*[tiab] OR “frailty syndrome”[tiab] OR “physical frailty”[tiab]) AND (“Aged”[Mesh] OR “Aged, 80 and over”[Mesh] OR older adult*[tiab] OR older people [tiab] OR elderly[tiab] OR aging[tiab] OR ageing[tiab] OR geriatric*[tiab]) AND (“Exercise Therapy”[Mesh] OR “Resistance Training”[Mesh]OR “Motor Activity”[Mesh]OR exercise[tiab] OR “physical activity”[tiab] OR “physical training”[tiab]OR “resistance training”[tiab] OR “strength training”[tiab]OR “multicomponent exercise”[tiab]OR aerobic[tiab]OR balance[tiab]OR rehabilitation[tiab]OR Baduanjin[tiab]OR exergam*[tiab])AND(“Diet Therapy”[Mesh]OR “Dietary Supplements”[Mesh]OR “Protein Supplementation”[tiab]OR “Nutritional Support”[tiab]OR nutrition[tiab] OR nutritional[tiab]OR diet*[tiab] OR protein[tiab] OR “protein supplementation”[tiab]OR “oral nutritional supplement*”[tiab]OR “amino acid*”[tiab]OR leucine[tiab]OR “branched-chain amino acid*”[tiab]OR BCAA[tiab]OR probiotic*[tiab]OR “milk fat globule membrane”[tiab]OR ginseng[tiab]OR “Panax ginseng”[tiab])AND(sarcopenia[tiab]OR “Sarcopenia”[Mesh]OR “muscle strength”[tiab]OR “muscle mass”[tiab]OR “physical performance”[tiab]OR “gait speed”[tiab]OR SPPB[tiab]OR “Short Physical Performance Battery”[tiab] OR “frailty status”[tiab]OR cognition[tiab]OR “quality of life”[tiab])AND(randomized controlled trial[pt]OR controlled clinical trial[pt]OR random*[tiab]OR trial[tiab]).

**Figure 1:**
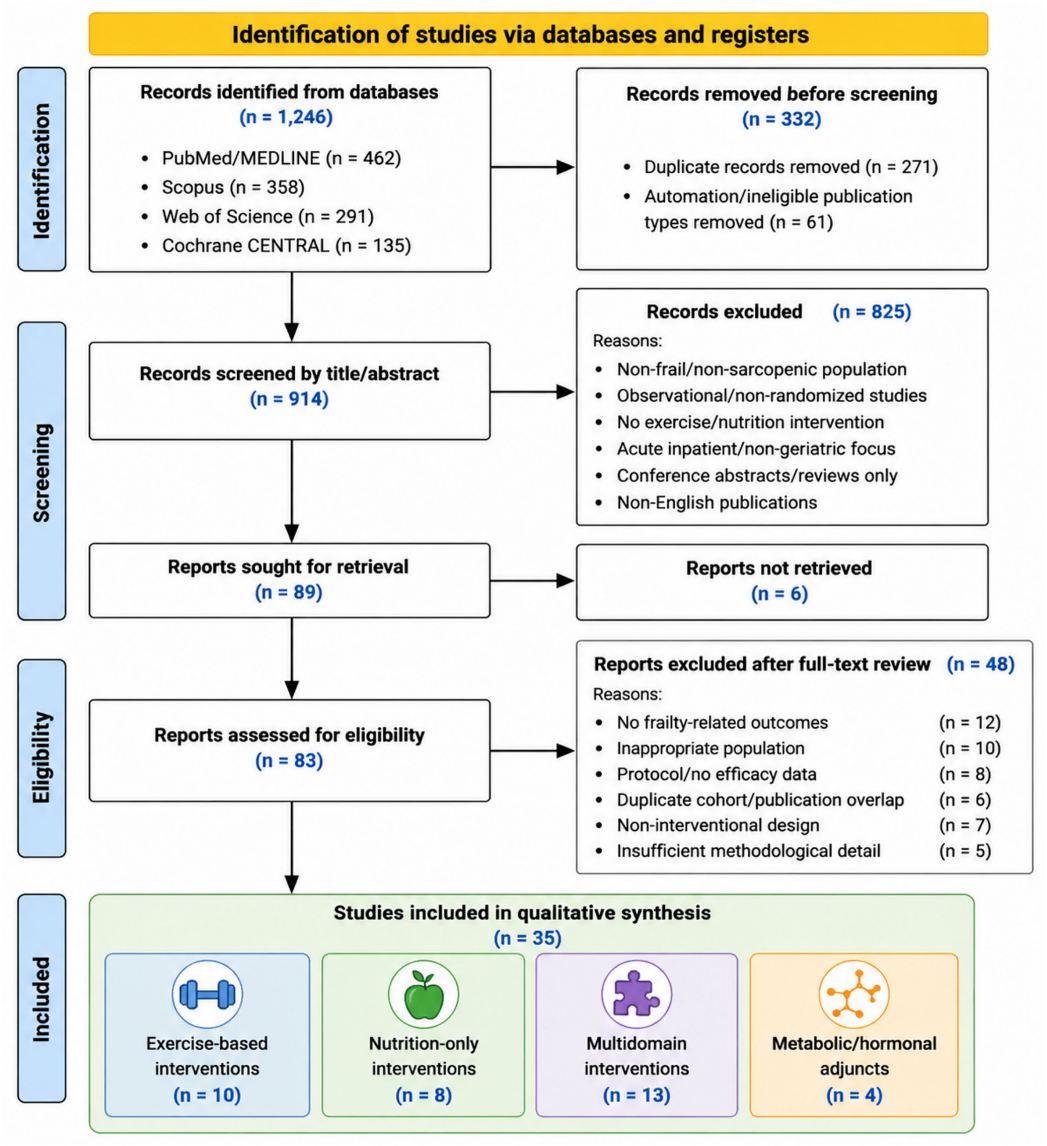
PRISMA 2020 flow diagram of study selection for the systematic review of exercise, nutritional, multidomain, and metabolic interventions targeting frailty and sarcopenia-related outcomes in older adults.

To maximize study identification, we manually screened the reference lists of included studies and relevant systematic reviews. Titles/abstracts and full texts were assessed against predefined eligibility criteria (population: frail or prefrail older adults; intervention: exercise, nutrition, or multidomain strategies; comparator: usual care or alternative intervention; outcomes: frailty-related and/or functional outcomes). Duplicate records were removed, and overlapping cohorts were checked; when multiple publications reported the same cohort, the most complete and/or most recent report was retained. The selection process is summarized in the PRISMA 2020 flow diagram (Figure 1 and Table 1) (25).

**Table 1:** Characteristics and outcomes of randomized controlled trials of exercise, nutrition, and multidomain interventions in frail or prefrail older adults.

**Study**	**Population (Frailty status)**	**Sample size**	**Intervention**	**Comparator**	**Duration**	**Primary frailty outcome**	**Physical function outcomes**	**Cognitive outcomes**	**Biomarkers / mechanistic outcomes**	**Main findings**
Singh et al. (1999) (40)	Frail older adults	26	Weight-lifting resistance training ± multinutrient supplementation	Placebo/control condition	10–14 weeks	↓ Sarcopenia-related muscle pathology	↑ Strength, ↑ Muscle fiber area, ↑ Type I trend	-	↑ IGF-1, ↑ Neonatal myosin, ↑ Muscle remodeling and ultrastructural adaptation	Robust anabolic and neuromuscular remodeling; strongest effects with combined exercise + nutrition
Tarazona-Santabalbina et al. (2016) (9)	Frail community-dwelling older adults	100	Multicomponent exercise (aerobic, strength, balance)	Usual care	24 w	Frailty phenotype reversal	↑ SPPB, Barthel, Tinetti	↑ MMSE	↓ Frailty biomarkers	Strong frailty reversal
Ng et al. (2015) (10)	Prefrail/frail community-	246	Nutritional, physical, cognitive, and combined multidomain interventions	Usual care	6–12 mo	↓ Frailty score	↑ Knee strength, gait speed, physical activity, energy	Not primary outcome	–	Combination intervention showed greatest frailty reversal
Ng et al. (2017) (21)	Prefrail/frail community	250	Exercise + nutrition + cognitive training	Usual care	6–12 mo	↓ Frailty score	↑ Gait speed, energy	↓ Depressive symptoms	–	Moderate improvement
Ng et al. (2018) (22)	Prefrail/frail with sarcopenia	92	Physical + cognitive + nutritional interventions	Control	6 mo	↓ Sarcopenia components	↑ Gait speed	↑ Cognition (training-specific)	–	Domain-specific effects
Lu et al. (2019) (38)	At-risk sarcopenic older adults	92	Multidomain lifestyle	Standard care	6 mo	↓ Sarcopenia prevalence	↑ Gait speed	No major change	–	Functional benefit
Yamada et al. (2015) (19)	Community older adults	227	Mail-based walking + nutrition	Control	6 mo	↓ Frailty risk	↑ SMI	-	↑ IGF-1, DHEA-S	Endocrine adaptation
Ye et al. (2024) (35)	Cognitive frailty	102	Baduanjin exercise	No exercise	24 w	↓ EFS	↑ Gait speed	↑ MoCA	↓ Oxidative stress, inflammation	Cognitive frailty improvement
Zak et al. (2009) (33)	Frail institutional + community	91	PRE + FOE ± nutrition	Standard exercise	7 w	Mixed frailty response	↑ Mobility OR strength	–	No clear biomarker data	Mode-specific effects
Tieland et al. (2012) (39)	Frail elderly	62	Resistance training + protein	Placebo	24 w	↓ Frailty trend	↑ SPPB	–	↑ Lean body mass	Synergistic effect
Park et al. (2018) (12)	Prefrail/frail malnourished	120	Protein supplementation (dose–response)	Lower protein dose	12 w	↓ sarcopenia risk	↑ Gait speed	–	↑ ASM	Dose-dependent
Miller et al. (2006) (14)	Post-fracture frail elderly	100	Resistance ± nutrition	Usual care	12 w	No reversal	No significant change	-	Weight loss prevention only	Limited functional gain
Payette et al. (2002) (31)	Community frail undernourished	83	Nutritional supplement	Usual care	16 w	No significant change	No strength change	–	↑ Weight gain	Nutrition-only limited
Niccoli et al. (2017) (13)	Hospitalized frail elderly	47	Whey protein supplementation	Control	Hospital stay	–	↑ Grip strength, knee force	–	↑ prealbumin correlation	Rehab support
Tabue-Teguo et al. (2018) (34)	Community-dwelling older adults	1,680	Multidomain intervention ± omega-3	Usual care / placebo	36 mo	No significant change	–	No significant cognitive benefit		No overall effect of multidomain or omega-3 on cognition; limited prefrail signal only
Román et al. (2024) (23)	Cirrhosis frailty	32	Exercise + BCAA + probiotics	Usual care	12 mo	↓ LFI	↓ Falls, ER visits	–	↓ CRP, LBP, sCD163	Strong clinical benefit
Laghi et al. (2024) (28)	Cirrhosis frailty	37	Exercise + BCAA + probiotics	Control	12 mo	↓ LFI	–	–	↓ Inflammation biomarkers	Systemic improvement
Vega-Abellaneda et al. (2025) (27)	Cirrhosis frailty	29	Same multifactorial	Control	12 mo	↓ LFI	–	–	Gut microbiome shift	Mechanistic support
Minetama et al. (2023) (18)	Postoperative elderly	80	BCAA + vitamin D	Placebo	12 w	No frailty change	↑ Knee strength	–	No body composition change	Partial anabolic effect
Rasmussen et al. (2024) (32)	Frail hypogonadal men	148	Testosterone + resistance training	Control	52 w	↓ Fatigue	↑ Chair stand	–	–	Synergistic benefit
Mone et al. (2022) (45)	Hypertensive frail elderly	72	L-arginine	Placebo	4 w	–	–	↑ MoCA	↓ Oxidative stress	Cognitive improvement
Kojima et al. (2023) (20)	Older adults at risk	120	MCT + exercise	Control	12 w	–	↑ Strength	–	Metabolic support	Additive effect
Solerte et al. (2008) (37)	Sarcopenic elderly	41	Amino acid supplementation	Placebo	18 mo	↓ Sarcopenia	↑ Lean mass	–	↓ TNF-α, ↑ IGF-1	Long-term anabolic effect
Taaffe et al. (1994) (47)	Elderly men	18	GH + resistance training	Placebo	10 w	No effect	No strength gain	–	↑ Lean mass only	No functional benefit
Lattanzi et al. (2021) (17)	Cirrhosis	24	HMB supplementation	Placebo	12 w	↓ LFI	↑ muscle function	–	–	Anabolic support
Latham et al. (2003) (24)	Frail older adults post-hospital discharge	243	Exercise + vitamin D	Placebo/usual visits	10 w	No improvemen	No functional benefit; ↑ injury risk	–	No vitamin D effect	Negative trial
Kim et al. (2015) (43)	Community-dwelling frail	130	Exercise + MFGM	Exercise + placebo	3 mo	Improved physical performance	↑ Strength, ↑ Body composition	–	Improved hematological and metabolic parameters	Synergistic benefits
Ninomiya et al. (2023) (48)	Frail older women post–THA	58	Exercise + protein/vitamin D	Exercise alone	12 w	No frailty endpoint	↑ Knee strength; no functional/QOL benefit	–	–	Limited additive benefit
Nagatomi et al. (2022) (29)	Frail patients with chronic heart failure	30	ICT-based home cardiac rehabilitation (exercise + nutrition + education)	Standard care	3 months	Improved frailty-related status	↑ 6MWD; ↑ lower-extremity strength	–	–	Home-based multidisciplinary intervention improved functional capacity
Park et al. (2023) (41)	Pre-frail older women	60	Protein-enriched meals + aerobic exercise ± electromyostimulation	Diet/control	8 weeks	↓ Frailty risk indicators	↑ Grip strength; ↑ SPPB; ↑ 6MWD	–	↑ HDL; ↑ endothelial function	Improved physical performance and vascular health
Rodrigues et al. (2022) (42)	Pre-frail/frail older adults	44	MoveStrong program (balance, strength training, protein education)	Stepped-wedge control	8 weeks	Trend toward frailty improvement	↑ Grip strength; ↑ sit-to-stand; ↑ balance	Trend toward improved HRQoL	–	Functional improvements support efficacy
Roschel et al. (2021) (44)	Pre-frail/frail older adults	200	Resistance training + protein/leucine/creatine supplementation	Placebo supplements	16 weeks	↔ No added frailty benefit	↑ Strength and function from exercise	–	↑ Lean mass	Exercise effective; supplements provided no additional benefit
Serra-Prat et al. (2022) (46)	Obese older adults at risk of frailty	305	Diet + physical activity weight-loss intervention	Usual care	24 months	↔ No significant frailty prevention	↑ Gait speed	–	↓ IL-6; ↓ insulin resistance; ↓ fat mass	Improved mobility and metabolic health despite no frailty reduction
Siramolpiwat et al. (2023) (30)	Frail patients with compensated cirrhosis	54	BCAA supplementation after diet and exercise counseling	Counseling only	16 weeks	↓ LFI; frailty reversal	↑ Skeletal muscle mass	–	↑ Albumin; ↑ SMI	Significant frailty reversal and improved quality of life
Travers et al. (2023) (36)	Community-dwelling older adults with pre-frailty/frailty	168	Home exercise + protein guidance (1.2 g/kg/day)	Usual care	3 months	↓ SHARE-FI frailty	↑ Grip strength	–	↑ Bone mass	Significant reduction in frailty prevalence

### Study selection

Titles and abstracts were screened for relevance to frailty, sarcopenia, exercise interventions, and nutritional supplementation. Full-text articles were then assessed for eligibility based on predefined inclusion criteria. Studies focusing exclusively on non-older populations, non-frailty outcomes, or non-interventional designs were excluded.

Eligible studies were categorized into three intervention groups:

1. Exercise-only interventions
2. Nutrition-only interventions
3. Combined/multidomain interventions (exercise + nutrition ± cognitive or behavioral components)

### Data extraction

Data were extracted from each included study using a standardized and structured extraction framework. Extracted variables included: study characteristics (author, year, country, and study design), population characteristics (sample size, mean age, frailty status and definition used, and clinical or community setting), intervention details (intervention domain[s], frequency, duration, intensity/progression, supervision level, and—for nutritional interventions—supplement composition, timing, and dosage), comparator description, and outcome measures.

To ensure transparent characterization of between-study variability relevant to synthesis, we also extracted features that reflected the diversity of experimental models and regimens, including: intervention modality (e.g., resistance training, multicomponent exercise, aerobic training, balance training, exergaming, traditional exercise forms), delivery model (home-based vs. supervised; individual vs. group), and clinical context (community-dwelling prefrail adults vs. hospitalized/post-acute, institutionalized, post-fracture/postoperative, or disease-specific cohorts such as cirrhosis). For nutritional strategies, we recorded the dosage regimen and formulation details (e.g., total protein targets, grams/day or g/kg/day where available; amino acid composition such as leucine/ BCAA enrichment; caloric content; co-interventions such as probiotics/omega-3/hormonal therapies; and supplementation duration), recognizing that these parameters differed substantially across trials.

Outcome data were extracted across multiple domains, including frailty status/indices, physical performance (e.g., gait speed, SPPB, chair-stand, balance and mobility tests), muscle strength (e.g., grip strength, knee extension strength), body composition (e.g., lean mass, appendicular skeletal muscle mass), cognitive outcomes (e.g., global cognition and domain-specific tests), and biomarkers (e.g., inflammatory markers, oxidative stress measures, anabolic/endocrine signals, and microbiome-related outcomes where reported). Main findings (direction of effect, statistical significance), as well as adverse events and safety concerns, were recorded. Where available, quantitative data (means/SDs, change scores, effect estimates, odds ratios, and confidence intervals) were extracted to support comparative narrative synthesis.

Given the breadth of extracted data, a meta-analysis was judged to be statistically unreliable because studies differed meaningfully in (i) experimental models and populations (baseline frailty severity, clinical stability, comorbidity burden, and setting), (ii) intervention “dose” and composition (exercise type/intensity/progression and supervision; nutrition formulation and protein dose; and frequent multidomain co-interventions), and (iii) outcome definitions and measurement approaches (multiple frailty instruments and thresholds, varied functional tests and follow-up intervals, and inconsistent reporting formats). These factors would have violated key assumptions required for valid quantitative pooling and risked producing a pooled estimate that was not clinically or statistically interpretable. Accordingly, extracted quantitative data were used to support structured comparison and narrative synthesis rather than formal aggregation.

### Outcome categorization

Outcomes were grouped into the following domains for synthesis:

1. Frailty outcomes: frailty phenotype status, frailty scores, LFI, frailty reversal rates
2. Physical performance: gait speed, SPPB, TUG
3. Muscle strength: grip strength, knee extension strength
4. Body composition: lean mass, skeletal muscle index, body weight changes
5. Cognitive outcomes: global cognition, memory, executive function
6. Biomarkers: inflammatory markers, anabolic hormones, oxidative stress indicators, microbiome composition
7. Safety outcomes: adverse events including musculoskeletal injury and tolerability

### Risk of bias assessment

Given the heterogeneity in reporting and the inclusion of both small pilot studies and larger RCTs, a formal meta-analysis was not conducted. Instead, methodological quality was assessed qualitatively across key domains, including randomization and allocation concealment procedures, blinding of participants and outcome assessors, completeness of outcome reporting, adequacy of sample size, and duration of follow-up. Studies identified as having a higher risk of bias—particularly those with limited blinding, small sample sizes, or incomplete reporting of outcomes—were interpreted with caution and given reduced weight in the overall synthesis of evidence.

### Data synthesis

A narrative synthesis approach was employed due to substantial heterogeneity across studies in frailty definitions and diagnostic criteria, intervention types and intensities, outcome measures, follow-up durations, and population characteristics and clinical settings. Findings were therefore synthesized thematically according to intervention categories (exercise-based, nutritional, and combined multidomain interventions) and outcome domains, including frailty status, physical function, muscle strength, cognition, biomarkers, and safety outcomes. Where feasible, consistent directional effects across studies were identified and summarized to highlight patterns of benefit or lack of effect. Particular emphasis was placed on the reproducibility and consistency of findings across independent trials rather than reliance on single-study results.

### Evidence certainty assessment

The certainty of evidence for each outcome domain was evaluated using the Grading of Recommendations Assessment, Development and Evaluation (GRADE) framework, which assesses the certainty of evidence across the domains of risk of bias, inconsistency, indirectness, imprecision, and publication bias (26). Each body of evidence was evaluated across these domains and synthesized to determine an overall certainty rating. The final certainty for each outcome was categorized as high, moderate, low, or very low certainty, based on the cumulative balance of strengths and limitations across the included studies (26).

### Summary of analytical approach

This synthesis integrates findings from randomized and controlled trials evaluating frailty-related interventions in older adults. The primary objective was to identify consistent patterns of effectiveness across intervention types, with particular emphasis on the comparative effectiveness of exercise, nutritional supplementation, and combined multidomain approaches in improving frailty and associated functional outcomes.

## Results

### Study characteristics

A total of 1,246 records were identified through database searching. After removal of 332 duplicate or ineligible records, 914 titles and abstracts were screened, of which 834 were excluded. Eighty full-text reports were sought for retrieval, and 74 articles were assessed for eligibility. Following full-text review, 39 articles were excluded for predefined reasons. Ultimately, 35 RCTs involving approximately 5,700 older adults were included in the qualitative synthesis (Figure 1).

The included studies evaluated exercise-based, nutrition-only, multidomain, and metabolic/hormonal interventions in frail, prefrail, sarcopenic, or at-risk older adults. Populations included community-dwelling, institutionalized, hospitalized, postoperative, and disease-specific cohorts, including individuals with cirrhosis, chronic heart failure, obesity, cognitive frailty, and hypogonadism (9, 10, 23, 27–32). Intervention duration ranged from 7 weeks to 36 months, although most studies lasted between 8 and 24 weeks (9, 33, 34).

### Effects on frailty outcomes

Frailty outcomes were reported in approximately 24 studies involving more than 4,300 participants. Exercise-based and multidomain interventions consistently improved frailty status across diverse populations, including reductions in frailty scores, frailty phenotype measures, Edmonton Frail Scale scores, SHARE-FI scores, and LFI measures (9, 10, 21–23, 28–30, 35, 36). The most consistent benefits were observed in interventions combining exercise with nutritional, educational, or cognitive components (9, 10, 21, 22, 29, 36).

Nutrition-only interventions generally produced less consistent effects on frailty despite improvements in nutritional status or body composition (12, 31, 37). Disease-specific multidomain programs in cirrhosis and chronic heart failure also demonstrated favorable effects on frailty-related outcomes and functional status (23, 28–30).

### Physical performance and muscle strength

Physical performance and muscle strength outcomes were reported in approximately 27 studies comprising more than 4,000 participants. Improvements in gait speed, mobility, balance, six-minute walk distance, SPPB scores, and functional independence measures were among the most consistent findings (9, 10, 21, 22, 29, 38–42). Resistance training consistently increased muscle strength, including grip strength and lower-extremity strength (39–43).

Combined exercise–nutrition interventions generally produced greater improvements than nutrition alone and were associated with favorable effects on mobility and physical function (18, 32, 39, 43). However, responses were less pronounced in recently hospitalized, postoperative, and post-fracture population (13, 31, 37).

### Muscle mass and body composition

Body composition outcomes were reported in approximately 15 studies involving more than 1,900 participants. Exercise combined with nutritional supplementation generally produced the greatest improvements in lean body mass, skeletal muscle mass, and body composition (12, 30, 39, 40, 44). Nutrition-only interventions improved body composition and nutritional biomarkers but often failed to produce parallel improvements in physical function or frailty outcomes (13, 31, 37).

Several studies reported anabolic adaptations, including increases in appendicular skeletal muscle mass, skeletal muscle index, serum albumin, and lean body mass (12, 30, 39, 44). Although several interventions increased muscle mass, these changes were not always associated with meaningful improvements in strength, mobility, or frailty status (44).

### Cognitive and psychosocial outcomes

Cognitive outcomes were assessed in approximately 8 studies involving more than 2,500 participants and showed greater heterogeneity than physical outcomes. Multidomain interventions and exercise-based programs demonstrated modest improvements in cognitive performance, particularly among participants with cognitive frailty (21, 22, 35, 45). Improvements in depressive symptoms and quality of life were also reported in some studies (21, 42).

However, evidence for cognitive benefit was inconsistent, and the largest multidomain trial reported no significant effect on cognition over long-term follow-up (34).

### Biomarkers and mechanistic outcomes

Biomarker or mechanistic outcomes were reported in approximately 15 studies involving more than 1,500 participants. Common findings included increases in anabolic markers such as insulin-like growth factor-1 (IGF-1), dehydroepiandrosterone sulfate (DHEA-S), serum albumin, and skeletal muscle index, alongside reductions in inflammatory and oxidative stress biomarkers (19, 23, 28, 30, 35, 37, 40, 44).

Additional studies reported improvements in endothelial function, insulin resistance, lipid profiles, and gut microbiome composition, suggesting potential anabolic, anti-inflammatory, metabolic, vascular, and microbiome-mediated mechanisms underlying frailty improvement (27, 30, 41, 46).

### Safety

Safety outcomes were reported in approximately 10 studies involving more than 1,200 participants. Most interventions were well tolerated, with few serious adverse events reported. Nutritional supplementation demonstrated a favorable safety profile (12, 13, 37). Exercise interventions were generally safe, although one study reported increased musculoskeletal injury risk following home-based resistance exercise in recently hospitalized frail adults (24). Hormonal interventions produced mixed findings, with testosterone improving functional outcomes whereas growth hormone increased lean mass without improving strength (32, 47).

### Risk of bias assessment

Overall methodological quality was moderate (Table 2). Most studies were judged as having some concerns regarding risk of bias, primarily related to incomplete reporting of randomization procedures, allocation concealment, and lack of participant blinding. No study was judged to be at high overall risk of bias. The frequent use of objective outcomes such as gait speed, SPPB, chair-stand performance, and grip strength reduced the likelihood of outcome measurement bias.

**Table 2:** Risk of bias assessment (cochrane RoB-style) of included randomized controlled trials.

**Study (Author, Year)**	**Randomization process**	**Deviations from intended interventions**	**Missing outcome data**	**Outcome measurement**	**Selective reporting**	**Overall risk**
Kojima et al. (20)	SC	SC	SC	L	SC	SC
Laghi et al. (28)	SC	SC	SC	SC	SC	SC
Latham et al. (24)	L	L	L	L	L	L
Lattanzi et al. (17)	SC	SC	SC	L	SC	SC
Lu et al. (38)	SC	SC	L	SC	SC	SC
Miller et al. (14)	L	SC	SC	L	SC	SC
Minetama et al. (18)	L	SC	L	L	SC	SC
Mone et al. (46)	SC	SC	SC	SC	SC	SC
Nagatomi et al. (29)	SC	SC	SC	SC	SC	SC
Ng et al. (10)	L	SC	SC	SC	SC	SC
Ng et al. (21)	L	SC	SC	SC	SC	SC
Ng et al. (22)	L	SC	SC	SC	SC	SC
Niccoli et al. (13)	L	SC	SC	SC	SC	SC
Ninomiya et al. (44)	SC	SC	SC	L	SC	SC
Park et al. (12)	L	SC	L	L	L	SC
Park et al. (41)	SC	SC	SC	L	SC	SC
Payette et al. (31)	SC	SC	SC	SC	SC	SC
Rasmussen et al. (32)	L	SC	SC	L	SC	SC
Rodrigues et al. (42)	SC	SC	SC	SC	SC	SC
Román et al. (23)	SC	SC	SC	SC	SC	SC
Roschel et al. (44)	L	SC	SC	L	SC	SC
Serra-Prat et al. (46)	L	SC	L	L	SC	SC
Singh et al. (40)	SC	SC	SC	SC	SC	SC
Siramolpiwat et al. (30)	SC	SC	SC	SC	SC	SC
Solerte et al. (37)	SC	SC	SC	SC	SC	SC
Taaffe et al. (47)	L	L	L	L	L	L
Tabue-Teguo et al. (34)	L	SC	SC	SC	SC	SC
Tarazona-Santabalbina et al. (9)	SC	SC	SC	L	SC	SC
Tieland et al. (39)	L	SC	L	L	L	SC
Travers et al. (36)	L	SC	SC	L	SC	SC
Vega-Abellaneda et al. (27)	SC	SC	SC	SC	SC	SC
Yamada et al. (19)	SC	SC	SC	SC	SC	SC
Ye et al. (35)	L	SC	SC	SC	SC	SC
Zak et al. (33)	SC	SC	SC	SC	SC	SC

Low risk (L); Some concerns (SC); High risk (H).

### Overall certainty of evidence

GRADE assessment indicated moderate-certainty evidence for exercise-based, combined exercise–nutrition, and multidomain interventions, reflecting consistent improvements in frailty status, muscle strength, and physical performance across randomized trials. Evidence for nutrition-only interventions was rated as low-to-moderate certainty because improvements in body composition were not consistently accompanied by functional benefits. Evidence for cognitive outcomes, biomarkers, and adjunct metabolic or pharmacologic therapies was generally low certainty because of heterogeneity in populations, interventions, and outcome measures (Table 3).

**Table 3:** GRADE evidence certainty for effects of exercise, nutritional, and multidomain interventions on frailty and sarcopenia outcomes in older adults.

**Outcome**	**No. of studies**	**Study design**	**Risk of bias**	**Inconsistency**	**Indirectness**	**Imprecision**	**Publication bias**	**Overall certainty**	**Summary of effect**
Frailty reversal / frailty improvement (LFI, SHARE-FI, CHS phenotype, EFS, Frailty Index)	16 RCTs	RCTs	Some concerns	Moderate	Low–moderate	Moderate	Possible	Moderate	Exercise-centered multidomain interventions consistently improve frailty status; strongest effects observed with exercise plus nutritional support
Muscle strength (grip strength, knee extension, chair stand, sit-to-stand)	20 RCTs	RCTs	Some concerns	Moderate	Low	Moderate	Unlikely	Moderate	Resistance and multicomponent exercise consistently improve strength; nutritional support may augment gains
Muscle mass / body composition (DEXA, BIA, SMI, lean mass)	16 RCTs	RCTs	Some concerns	Moderate–high	Low–moderate	Moderate	Possible	Moderate	Protein, amino acids, BCAA, testosterone, and exercise improve lean mass, although translation to function is variable
Physical performance (SPPB, gait speed, TUG, 6MWT, Barthel Index)	18 RCTs	RCTs	Some concerns	Moderate	Low	Moderate	Unlikely	Moderate	Consistent improvements in mobility, gait speed, balance, and physical performance across exercise-based interventions
Cognitive outcomes (MoCA, MMSE, cognitive frailty measures)	8 RCTs	RCTs	Some concerns	High	Moderate	Serious	Possible	Low	Multidomain interventions may improve cognition in cognitively frail populations, but findings remain inconsistent
Depressive symptoms / psychosocial wellbeing / HRQoL	5 RCTs	RCTs	Some concerns	Moderate	Moderate	Moderate	Possible	Low–Moderate	Exercise and multidomain interventions may improve mood, quality of life, and psychosocial functioning
Hospitalization, falls, healthcare utilization, caregiver burden	7 RCTs	RCTs	Some concerns	Moderate	Moderate	Moderate	Possible	Low–Moderate	Evidence suggests reductions in falls, emergency visits, and caregiver burden in selected populations, particularly cirrhosis and institutional care settings
Biomarkers and mechanistic outcomes (IGF-1, IL-6, TNF-α, CRP, oxidative stress, insulin resistance, microbiome, glycolysis)	12 RCTs	RCTs	Some concerns	High	Serious (surrogate outcomes)	Moderate	Possible	Low	Consistent biological signals support anabolic, anti-inflammatory, metabolic, vascular, endocrine, and microbiome-mediated mechanisms, but clinical significance remains uncertain
Quality of life / functional independence (ADL, Barthel, SF-36, HRQoL)	9 RCTs	RCTs	Some concerns	Moderate	Low	Moderate	Possible	Moderate	Exercise and multidomain interventions improve functional independence and health-related quality of life
Disease-specific frailty outcomes (cirrhosis-related frailty, heart failure frailty)	5 RCTs	RCTs	Some concerns	Moderate	Moderate	Moderate	Possible	Moderate	Multicomponent interventions improve frailty and functional outcomes in chronic disease populations, particularly cirrhosis and heart failure

## Discussion

This systematic review demonstrates that exercise-centered and multidomain interventions represent the most effective strategies for improving frailty, sarcopenia, and functional decline in older adults. Across diverse clinical and community settings, interventions combining resistance or multicomponent exercise with targeted nutritional support consistently produced superior improvements in muscle strength, physical performance, and frailty status compared with single-component interventions alone. Recent studies further strengthen this conclusion. For example, Nagatomi et al. (29) showed that an information and communication technology (ICT)-based home cardiac rehabilitation program integrating exercise, nutrition counseling, and education significantly improved exercise capacity and lower-extremity strength among frail patients with chronic heart failure. Similarly, Park et al. (41) demonstrated that protein-enriched meals combined with aerobic exercise improved grip strength, SPPB scores, and six-minute walk distance in pre-frail older women, while also enhancing vascular function. These findings reinforce the concept that frailty is a multidimensional and potentially reversible syndrome driven by interconnected physiological, metabolic, inflammatory, and behavioral mechanisms.

A major finding of this review is the central role of exercise—particularly progressive resistance and multicomponent training—as the primary driver of functional improvement. Studies such as Tarazona-Santabalbina et al. (9), Tieland et al. (39), Singh et al. (40), Rodrigues et al. (42), and Nagatomi et al. (29) demonstrated substantial gains in muscle strength, gait speed, balance, chair-stand performance, and functional independence following structured exercise interventions. The MoveStrong program evaluated by Rodrigues et al. (42) produced significant improvements in grip strength, sit-to-stand performance, and dynamic balance among pre-frail and frail older adults, supporting the feasibility and effectiveness of community-based exercise programs. Collectively, these findings support the growing consensus that neuromuscular stimulation remains the most effective strategy to counteract age-related declines in muscle quality and mobility. Importantly, improvements were observed even among highly frail and clinically vulnerable populations, indicating that advanced age and frailty do not eliminate responsiveness to anabolic and functional adaptation.

The mechanistic findings reported by Singh et al. (40) remain particularly important in understanding the biological basis of frailty reversal. Their observation of marked increases in skeletal muscle IGF-1 expression, neonatal myosin staining, and muscle fiber hypertrophy following resistance exercise suggests that frail skeletal muscle retains substantial regenerative plasticity. These results challenge earlier assumptions that frailty-related sarcopenia is largely irreversible. Instead, the data indicate that anabolic signaling pathways remain responsive to mechanical loading, especially when adequate nutritional support is provided.

Nevertheless, exercise alone was not uniformly effective across all populations. Several studies demonstrated that functional improvements were attenuated in highly vulnerable clinical populations, such as post-hospitalized or postoperative older adults. For example, Latham et al. (24) reported no significant improvements in falls or physical performance following home-based quadriceps resistance exercise, and participants experienced increased musculoskeletal injury risk. Similarly, Miller et al. (14) found limited functional gains in frail post-fracture patients despite nutritional and resistance interventions. These findings suggest that intervention responsiveness may depend heavily on baseline frailty severity, acute illness burden, recovery phase, and exercise supervision. Frail individuals recovering from hospitalization may require gradual progression, individualized dosing, and closer monitoring to avoid adverse events and optimize adherence.

The review also highlights the complementary but variable role of nutritional supplementation in supporting anabolic adaptation. Nutrition-only interventions generally improved energy intake, body weight, or lean mass but often failed to produce clinically meaningful functional gains when implemented without exercise. For instance, Payette et al. (31) demonstrated improved caloric intake and weight gain without significant improvements in strength or mobility. Likewise, Serra-Prat et al. (46) reported significant reductions in body weight, fat mass, insulin resistance, and inflammatory markers, together with improved gait speed, but found no significant effect on frailty prevention over 24 months. These findings suggest that metabolic improvements alone may be insufficient to reverse frailty without concurrent improvements in neuromuscular function.

This apparent dissociation between muscle mass and physical function is clinically important. Increasing lean mass alone may not translate into meaningful improvements in mobility or frailty unless accompanied by neuromuscular stimulation through exercise. Muscle quality, neural recruitment, coordination, balance, and cardiorespiratory reserve likely contribute as much to frailty outcomes as absolute muscle quantity. This concept may help explain why several supplementation trials demonstrated favorable anabolic responses without corresponding improvements in functional performance.

However, when nutritional supplementation was combined with exercise, synergistic effects became more apparent. Tieland et al. (39) showed that protein supplementation augmented lean mass gains during resistance training, while Park et al. (12) demonstrated dose-dependent improvements in appendicular muscle mass and gait speed with higher protein intake. More recent studies provide additional support. Travers et al. (36) found that a home-based strength-focused exercise program combined with dietary protein guidance significantly reduced frailty prevalence and improved grip strength and bone mass, with a number needed to treat of approximately eight. Park et al. (41) similarly reported improvements in strength, physical performance, and vascular function when protein-enriched meals were combined with exercise. Siramolpiwat et al. (30) demonstrated that BCAA supplementation following dietary and exercise counseling significantly improved LFI scores, skeletal muscle mass, serum albumin levels, and quality of life among frail patients with compensated cirrhosis. Collectively, these studies support the hypothesis that adequate protein availability enhances the adaptive response of aging skeletal muscle to exercise stimuli.

At the same time, recent evidence suggests that the benefits of supplementation may depend on the specific intervention context. Roschel et al. (44) reported that whey protein, soy protein, leucine, and creatine supplementation failed to provide additional benefits beyond those achieved through resistance training alone in pre-frail and frail older adults. Despite improvements in muscle mass and strength across all groups, supplementation did not augment training-induced adaptations. These findings indicate that exercise remains the primary therapeutic driver, whereas nutritional supplementation may be most beneficial in individuals with nutritional deficiencies, inadequate protein intake, or specific clinical conditions.

Protein dose also appears to be a critical determinant of response. The findings by Park et al. (12) suggest that protein intakes approaching 1.5 g/kg/day may be necessary to optimize anabolic and functional outcomes in frail older adults, exceeding traditional dietary recommendations for healthy aging populations. Similarly, Travers et al. (36) employed a target protein intake of approximately 1.2 g/kg/day and observed significant reductions in frailty prevalence. These observations align with emerging geriatric nutrition guidelines advocating higher protein intake thresholds in sarcopenic and frail populations. However, variability in nutritional status, renal function, and comorbidities necessitates individualized nutritional prescriptions.

An important contribution of this review is the growing evidence supporting multidomain interventions. Studies integrating exercise, nutrition, cognitive stimulation, psychosocial support, or educational components consistently demonstrated broader and more clinically meaningful benefits than isolated interventions. Ng et al. (10, 21, 22) showed that multidomain programs improved frailty scores, physical performance, mood, and selected cognitive outcomes. Tarazona-Santabalbina et al. (9) reported improvements not only in physical function but also in cognition, emotional status, and social engagement. Likewise, Nagatomi et al. (29) demonstrated benefits from a multidisciplinary home-based program combining exercise, nutrition counseling, and education, while Rodrigues et al. (42) integrated strength training with nutritional education to achieve meaningful functional gains.

These findings reinforce the conceptualization of frailty as a systemic and multidimensional syndrome rather than solely a musculoskeletal disorder. Frailty encompasses physical, cognitive, psychological, inflammatory, metabolic, and social vulnerability domains that interact bidirectionally. Consequently, interventions targeting multiple pathways simultaneously may produce additive or synergistic effects that exceed those achievable through isolated approaches. The multidomain model also aligns closely with contemporary geriatric care frameworks emphasizing integrated, personalized, and function-centered management strategies.

Evidence from cirrhosis populations further strengthens the multidimensional interpretation of frailty. Román et al. (23), Siramolpiwat et al. (30), Vega-Abellaneda et al. (27), and related studies demonstrated that combinations of exercise, BCAA supplementation, and probiotic therapy improved LFI scores while simultaneously influencing inflammatory pathways, nutritional status, muscle mass, and gut microbiome composition. These findings are particularly significant because they suggest that frailty may be partially mediated through systemic inflammatory and metabolic pathways involving the gut–liver–muscle axis. Observed reductions in inflammatory markers and favorable microbiome remodeling provide preliminary evidence that anti-inflammatory and metabolic modulation may contribute to functional recovery.

The emerging role of the gut microbiome in frailty biology represents an important area for future investigation. Age-related dysbiosis, chronic low-grade inflammation, altered amino acid metabolism, and impaired mitochondrial function have all been implicated in sarcopenia and frailty pathogenesis. The cirrhosis studies included in this review provide early mechanistic evidence that microbiome-targeted interventions may influence frailty trajectories, although causality remains uncertain.

Cognitive outcomes across studies were more heterogeneous and generally less robust than physical outcomes. Exercise and multidomain interventions produced modest improvements in cognition, particularly among cognitively frail populations. Ye et al. (35) demonstrated improvements in both frailty and Montreal Cognitive Assessment (MoCA) scores following Baduanjin exercise, accompanied by reductions in oxidative stress and inflammatory markers. Mone et al. (45) similarly observed improvements in cognitive performance following L-arginine supplementation. In contrast, Tabue-Teguo et al. (34)found no significant cognitive benefit from multidomain intervention or omega-3 supplementation in the Multidomain Alzheimer Preventive Trial (MAPT) cohort. Several factors may explain this inconsistency, including longer timelines for cognitive change, differences in baseline cognitive status, and variability in cognitive assessment methods. Nevertheless, the observed links among physical function, inflammation, oxidative stress, and cognition support the concept of cognitive frailty as a biologically interconnected syndrome.

Hormonal and metabolic adjunct therapies produced mixed findings. Testosterone combined with resistance training improved fatigue and physical performance in hypogonadal frail men (32), whereas growth hormone supplementation failed to augment strength gains despite increasing lean mass (47). These findings suggest that isolated endocrine manipulation may not overcome the complex neuromuscular and inflammatory processes underlying frailty. Furthermore, hormonal therapies raise concerns regarding safety, cardiovascular risk, cost, and long-term feasibility in older populations. Overall, current evidence suggests that hormonal therapies may be most effective when used as adjuncts to structured exercise and nutritional interventions rather than as stand-alone treatments.

Taken together, the expanded evidence base increasingly supports a hierarchical model of frailty intervention in which exercise serves as the essential therapeutic foundation, nutritional optimization enhances responsiveness to exercise, and multidomain approaches provide the greatest potential for durable improvements in frailty, physical function, and overall resilience. Recent studies published between 2021 and 2025 largely reinforce this paradigm while highlighting the importance of individualized intervention strategies tailored to frailty severity, comorbidity burden, nutritional status, and underlying disease mechanisms.

### Clinical implications

The findings of this review have important implications for the prevention and management of frailty and sarcopenia in aging populations. First, the evidence consistently supports structured exercise, particularly progressive resistance and multicomponent exercise training, as the cornerstone intervention for improving muscle strength, mobility, and frailty status in older adults. Clinicians should therefore prioritize exercise prescription as a routine component of geriatric care rather than viewing frailty solely as an unavoidable consequence of aging.

Second, the review highlights the importance of combining exercise with targeted nutritional support, especially adequate protein or amino acid supplementation, to optimize anabolic responses and preserve skeletal muscle mass. Nutritional interventions alone may improve body weight or lean mass, but their functional benefits appear limited without concurrent physical training. These findings support integrated exercise–nutrition programs for frail, prefrail, and sarcopenic older adults, particularly in individuals with low protein intake, malnutrition risk, or recent hospitalization.

Third, the evidence suggests that frailty interventions should be individualized according to baseline frailty severity, comorbidities, cognitive status, and clinical setting. Highly frail or post-acute patients may require lower initial exercise intensity, closer supervision, and gradual progression to minimize adverse events and improve adherence. In contrast, community-dwelling prefrail individuals may benefit from earlier preventive multidomain interventions before severe disability develops.

Fourth, multidomain strategies integrating exercise, nutrition, cognitive stimulation, and psychosocial support demonstrated broader benefits across physical, cognitive, and emotional domains. These findings reinforce the need for multidisciplinary geriatric care models involving physicians, physiotherapists, exercise professionals (e.g., exercise physiologists or certified exercise specialists), dietitians, nurses, and community health professionals. Incorporating frailty screening and intervention into primary care, rehabilitation, and long-term care settings may help delay disability progression and preserve independence.

The emerging evidence linking frailty improvement with reductions in inflammation, oxidative stress, and gut microbiome alterations also suggests potential opportunities for biologically targeted interventions in the future. Although mechanistic findings remain preliminary, they support the concept that frailty is a modifiable systemic condition rather than solely a musculoskeletal disorder.

From a public health perspective, effective frailty interventions may reduce falls, hospitalization, institutionalization, healthcare utilization, and caregiver burden. Community-based and home-based exercise and nutrition programs could provide scalable and cost-effective strategies to address the growing burden of frailty in aging societies. However, successful implementation will require attention to adherence, accessibility, long-term sustainability, and individualized care delivery.

### Limitations of the evidence base

Several limitations should be considered when interpreting the findings of this review. A formal meta-analysis was not conducted because pooling was not methodologically defensible given the degree of clinical and methodological heterogeneity across trials. Specifically, studies differed in (1) how frailty was defined and operationalized (e.g., phenotype-based criteria, gait speed thresholds, sarcopenia-related indices, or disease-specific tools), meaning participants were not comparable across trials; (2) intervention characteristics (exercise modality, intensity, frequency, supervision, progression; and nutritional composition/dose/timing), resulting in non-equivalent treatment “exposures”; and (3) outcome selection and measurement, with diverse endpoints (physical performance, strength, body composition, cognition, Quality of Life (QoL), biomarkers) assessed using different instruments and at different follow-up timepoints. Under these conditions, a pooled estimate would risk combining fundamentally different populations, interventions, and outcomes and could produce a summary effect that is misleading rather than informative. Therefore, the review relied on narrative synthesis emphasizing within-study effects and directional consistency.

Many included trials also had methodological limitations that may have introduced bias. Blinding was often not feasible in exercise-based interventions, and reporting of allocation concealment, randomization procedures, and assessor blinding was inconsistent. Several studies had small sample sizes, limited statistical power, and incomplete reporting of attrition or intention-to-treat analyses, reducing confidence in the precision and generalizability of findings.

Intervention protocols were heterogeneous and variably described. Exercise interventions differed in modality, intensity, frequency, progression, and supervision, while nutritional interventions varied in composition, dose, timing, and duration. Adherence, intervention fidelity, and co-interventions were also inconsistently reported, limiting causal interpretation and reproducibility. Outcome selection represents another important limitation. Although many studies reported improvements in surrogate or intermediate outcomes, fewer assessed clinically meaningful endpoints such as falls, hospitalization, disability progression, institutionalization, healthcare utilization, or mortality. Follow-up periods were often short, making it difficult to determine the durability and long-term clinical relevance of observed benefits. Population heterogeneity further limits external validity, as participants ranged from relatively healthy prefrail community-dwelling adults to hospitalized, institutionalized, postoperative, and cirrhotic patients with advanced frailty and multimorbidity. Publication bias and single-center study designs also cannot be excluded. Finally, mechanistic findings involving inflammatory, endocrine, oxidative stress, or microbiome pathways should be interpreted as exploratory, as most studies were not designed to test mediation or establish causal pathways.

### Future research directions

Future research should focus on improving the methodological rigor, standardization, and translational applicability of frailty intervention studies. One of the most important priorities is the development of standardized frailty definitions and outcome measures. The current literature is limited by substantial heterogeneity in diagnostic criteria, including use of the Fried phenotype, sarcopenia indices, gait speed thresholds, and disease-specific frailty tools. Greater harmonization of frailty assessment methods would improve comparability across studies and facilitate future meta-analyses and evidence synthesis.

Large-scale, multicenter RCTs with longer follow-up durations are also needed. Many existing studies were small, short-term, or exploratory in nature, limiting statistical power and long-term interpretation. Future trials should evaluate clinically meaningful endpoints beyond surrogate measures of muscle mass or strength, including hospitalization, falls, disability progression, institutionalization, healthcare utilization, quality of life, and mortality. Longitudinal studies examining sustainability of intervention effects after program completion are particularly important.

Further investigation is required to determine the optimal type, intensity, frequency, and duration of exercise interventions for different frailty phenotypes. Although resistance and multicomponent exercise consistently demonstrated benefits, the ideal “dose” of exercise for highly frail, hospitalized, postoperative, or cognitively impaired populations remains unclear. Personalized or adaptive exercise prescriptions based on baseline frailty severity, mobility status, and comorbidity burden should be explored.

Similarly, future nutritional research should aim to establish evidence-based recommendations for protein intake, amino acid composition, supplementation timing, and combined nutritional strategies in frail older adults. While higher protein intake and amino acid supplementation appear promising, the threshold required to optimize anabolic response may differ according to age, disease status, inflammation, renal function, and baseline nutritional status. Research examining nutrient timing in relation to exercise and muscle protein synthesis may further refine intervention efficacy.

The growing evidence supporting multidomain interventions warrants additional investigation into integrated care models combining exercise, nutrition, cognitive training, psychosocial support, and behavioral interventions. Future studies should determine which intervention components provide the greatest additive or synergistic effects and identify strategies that maximize long-term adherence and implementation in real-world settings.

Mechanistic and translational research also represents a major future priority. Emerging evidence suggests that frailty may be influenced by inflammatory signaling, oxidative stress, endocrine dysregulation, mitochondrial dysfunction, and alterations in gut microbiome composition. Further studies integrating omics technologies, metabolomics, microbiome analysis, and biomarker profiling could improve understanding of frailty biology and help identify therapeutic targets or predictors of intervention responsiveness.

In particular, the role of the gut–muscle axis and microbiome-targeted therapies deserves greater exploration. Preliminary evidence from cirrhosis populations suggests that probiotics and multidomain interventions may influence frailty through modulation of inflammation and microbial composition. Whether similar mechanisms operate in broader geriatric populations remains uncertain and should be evaluated in future translational trials.

Research should also prioritize underrepresented populations, including very old adults (>85 years), ethnically diverse populations, institutionalized individuals, and those with multimorbidity or cognitive impairment. Many current trials primarily involve relatively healthy community-dwelling older adults, limiting generalizability to more vulnerable populations with advanced frailty.

Implementation science approaches are equally important. Future studies should examine cost-effectiveness, scalability, digital health integration, tele-rehabilitation, adherence strategies, and healthcare system implementation to support broader adoption of frailty interventions in clinical and community practice. Home-based and technology-assisted interventions may become increasingly important as aging populations expand globally.

Finally, precision medicine approaches may help optimize frailty management in the future. Identifying biological, functional, nutritional, or psychosocial predictors of treatment response could enable more individualized intervention strategies tailored to specific frailty phenotypes and patient characteristics. Such approaches may improve effectiveness while minimizing unnecessary intervention burden in vulnerable older adults.

## Conclusion

This systematic synthesis of randomized controlled and controlled clinical trials indicates that interventions targeting frailty in older adults are most effective when they combine exercise with nutritional or multidomain strategies. Across heterogeneous populations, including community-dwelling, institutionalized, postoperative, and disease-specific cohorts, exercise-based interventions consistently improved muscle strength and physical performance, while nutritional supplementation primarily enhanced muscle mass and anabolic markers, particularly when protein intake was adequate or combined with resistance training.

Multidomain interventions integrating physical activity with nutrition and cognitive training produced the most consistent benefits on frailty status, functional outcomes, and, in some cases, cognitive and psychosocial domains. In contrast, nutrition-only strategies showed limited and inconsistent effects on functional frailty reversal, despite improving energy intake and body composition. Biomarker evidence suggests that observed clinical improvements are likely mediated through anabolic, anti-inflammatory, and metabolic pathways, although mechanistic findings remain exploratory.

Importantly, effects were not uniform across all populations; frail individuals in acute or highly vulnerable clinical settings showed attenuated responses, and isolated pharmacological or hormonal interventions demonstrated inconsistent functional benefit. Safety signals were generally favorable, although high-intensity unsupervised exercise may increase adverse events in selected frail populations.

Overall, the evidence supports combined exercise and nutritional approaches as the most effective and clinically relevant strategy for mitigating frailty and sarcopenia in older adults. Future research should prioritize standardized frailty definitions, longer follow-up periods, and adequately powered trials to clarify optimal intervention dose, composition, and implementation in real-world healthcare settings.

## Data Availability

The original contributions presented in the study are included in the article/supplementary material, further inquiries can be directed to the corresponding author/s.
